# Molecular timetrees using relaxed clocks and uncertain phylogenies

**DOI:** 10.3389/fbinf.2023.1225807

**Published:** 2023-08-03

**Authors:** Jose Barba-Montoya, Sudip Sharma, Sudhir Kumar

**Affiliations:** ^1^ Institute for Genomics and Evolutionary Medicine, Temple University, Philadelphia, PA, United States; ^2^ Department of Biology, Temple University, Philadelphia, PA, United States

**Keywords:** relaxed molecular clock, phylogenetic uncertainty, phylogenomics, bootstrap, timetree

## Abstract

A common practice in molecular systematics is to infer phylogeny and then scale it to time by using a relaxed clock method and calibrations. This sequential analysis practice ignores the effect of phylogenetic uncertainty on divergence time estimates and their confidence/credibility intervals. An alternative is to infer phylogeny and times jointly to incorporate phylogenetic errors into molecular dating. We compared the performance of these two alternatives in reconstructing evolutionary timetrees using computer-simulated and empirical datasets. We found sequential and joint analyses to produce similar divergence times and phylogenetic relationships, except for some nodes in particular cases. The joint inference performed better when the phylogeny was not well resolved, situations in which the joint inference should be preferred. However, joint inference can be infeasible for large datasets because available Bayesian methods are computationally burdensome. We present an alternative approach for joint inference that combines the bag of little bootstraps, maximum likelihood, and RelTime approaches for simultaneously inferring evolutionary relationships, divergence times, and confidence intervals, incorporating phylogeny uncertainty. The new method alleviates the high computational burden imposed by Bayesian methods while achieving a similar result.

## 1 Introduction

Most molecular systematics studies reporting species divergence times currently apply relaxed molecular clock dating to a specified phylogeny (dos Reis et al., 2016; Tao et al., 2020). However, all evolutionary relationships in a molecular phylogeny rarely receive high statistical support, even for phylogenomic datasets (Kapli et al., 2020; Sharma and Kumar, 2021). In this case, the sequential practice of first inferring phylogeny and then estimating divergence times is expected to cause overconfidence in the estimates of some divergence times, i.e., narrower confidence and credibility intervals (Cranston and Rannala, 2005; Thorne and Kishino, 2005; Ho and Phillips, 2009; Lee et al., 2009; Ronquist et al., 2012a). Consequently, the joint inference of phylogeny and divergence time is advocated (Cranston and Rannala, 2005; Drummond et al., 2006; Sauquet, 2013; Bromham et al., 2018). For example, Bayesian methods consider many likely tree topologies and build a posterior distribution of divergence times (Ronquist et al., 2012b; Drummond et al., 2012; Hohna et al., 2016; Bouckaert et al., 2019).

Although there is a general belief that phylogenetic uncertainty may impact time estimates and credibility intervals (Drummond et al., 2006; Ho and Phillips, 2009; Ronquist et al., 2012a; Sauquet, 2013; Ho and Duchêne, 2014; Bromham et al., 2018), there is little information regarding the accuracy gains achievable by jointly inferring both phylogeny and divergence times. In fact, the impact of topological uncertainty on divergence times may be limited to branches with short duration (Yoder and Yang, 2000; Thorne and Kishino, 2005). Therefore, one major objective of this study was to use empirical and simulated datasets to quantify accuracy gains afforded by the joint inference of evolutionary relationships and divergence time estimates.

**FIGURE 1:**
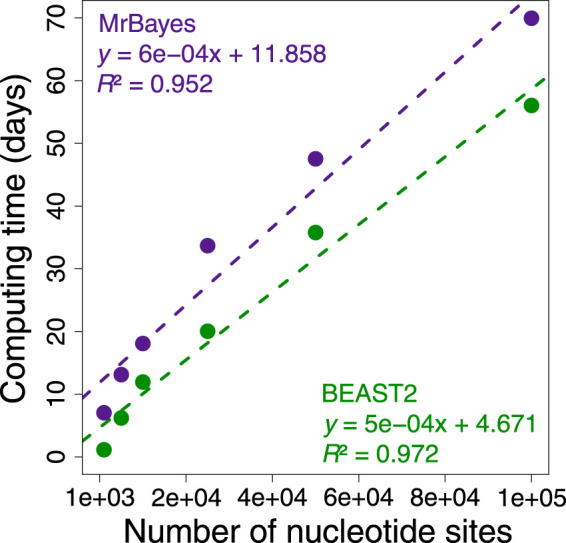
Comparison of BEAST2 (green dots) and MrBayes (purple dots) computing times for analysis of subsets of 1,000, 5,000, 10,000, 25,000, 50,000, and 100,000 nucleotide sites from a concatenation of 72 mammal sequences and 33,173,174 sites form the study by Álvarez-Carretero et al. (2022). We calculated the computing time required for each analysis to reach a minimum effective sample size of 200 for all the parameters using only one thread. The equations and coefficient of determination (*R* ^2^) for the linear regressions are shown. The dashed lines represent the best-fit linear regression for BEAST2 (green) and MrBayes (purple).

During these investigations, we found that applying Bayesian methods to infer phylogeny and times for phylogenomic datasets jointly was too time consuming because the complexity of the underlying likelihood calculations increases with sequence length and the number of taxa (Sharma and Kumar, 2021). This is evident from a linear escalation in the computational time required to analyze increasingly longer subsets of a phylogenomic dataset of 72 mammalian species and 33,173,174 nucleotide sites (Figure 1). This trend predicted that Bayesian analysis would require more than 49 years of computing time if we were to analyze the whole dataset proposed by Álvarez-Carretero et al. (2022) at one time (Supplementary Table S1). For this reason, many investigators resort to using data subsamples and combining the estimated dates (Jetz et al., 2012; Tonini et al., 2016; dos Reis et al., 2018; Jetz and Pyron, 2018; Upham et al., 2019; Álvarez-Carretero et al., 2022). These divide-and-conquer approaches effectively decrease computational times but often require sequential analysis to estimate times.

Here, we also present a bootstrap phylogeny approach for jointly inferring phylogeny and times for large datasets of tens of thousands to millions of sites. Our method uses the bag of little bootstraps method (LBS) framework (Sharma and Kumar 2021) that analyzes tiny subsamples of site patterns to decrease the computational time and memory needs by orders of magnitude. Our method uses the little bootstraps method resampling method to generate alternative phylogenies, each subjected to relaxed clock dating using the relative rate framework (Tamura et al., 2012). The resulting little bootstrap replicate timetrees are then used to produce a consensus phylogeny, divergence times, and confidence intervals that automatically incorporate phylogenetic uncertainty. We use the maximum likelihood (ML) approach to infer phylogenies and estimate branch lengths. We also explored using the standard bootstrap resampling (BS) method (Felsenstein, 1985) for small datasets that may contain hundreds to thousands of sites.

We present our new approach and compare its performance with Bayesian methods using empirical and computer-simulated datasets in the following section. Specifically, we focus on inferred phylogenies containing many clades with low statistical support, addressing gaps in our knowledge about the usefulness of joint inference and the need for computationally efficient methods for bigger datasets. We focused our investigation on dating analyses in which no time constraints on internal nodes were applied, except for a single ingroup root calibration. This choice allowed us to directly examine the power of both methods in dealing with phylogenetic uncertainty without using internal calibrations that are expected to make results from joint analysis (JA) and sequential analysis (SA) more similar. The root was specified in all the analyses because Bayesian methods may produce biased times when the root is required to be inferred, and the specification of an ingroup clade is a requirement in RelTime.

## 2 Materials and methods

### 2.1 A new joint inference (JA) approach

**FIGURE 2:**
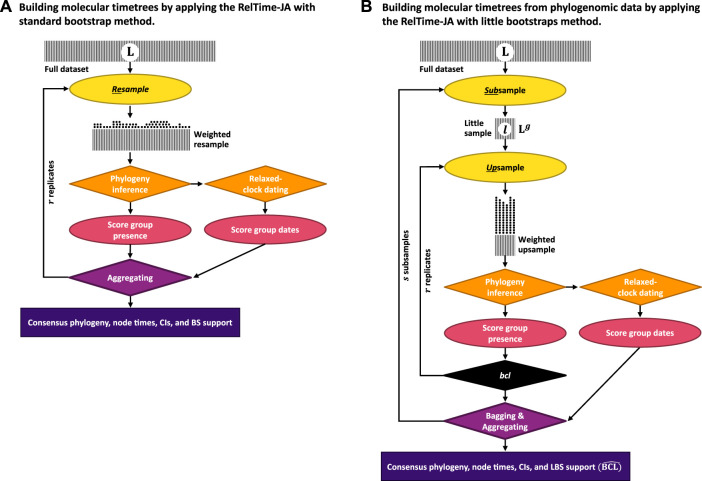
Steps in the **(A)** RelTime-JA with standard bootstraps and **(B)** RelTime-JA with little bootstraps method. Shaded boxes represent sequence alignments, with width representing the sequence length. In **(A)** RelTime-JA with standard bootstraps, L sites are randomly sampled with replacement from the original dataset containing L sites. In this resampling process, a proportion of the data points are expected to be represented in a bootstrap replicate dataset. Each replicate dataset is compressed into weighted resamples that contain only distinct site configurations and a vector of their counts (represented by stacks of dots). An ML tree is inferred from each replicate dataset, and the BS support for a node/clade is the proportion of times that appeared in bootstrap replicate phylogenies. Each ML tree is dated using RelTime to generate node ages and CIs; then, time estimates are summarized on the BS consensus tree. In **(B)** RelTime-JA with little bootstraps, L sites are randomly sampled with replacement from the little dataset consisting of only l = L^*g*^ sites, which produces bootstrap replicate datasets. This procedure automatically determines the size of little samples (*l*) by adjusting the power factor (*g*). Power factor (*g*) estimation is given in the study by Sharma and Kumar (2021). Because l ≪ L, each site will be represented many times in the LBS replicate datasets, which we refer to as upsampling that changes the frequency of distinct site configurations. Stacks of dots are much higher for LBS due to upsampling than for BS, which involves only resampling. The number of distinct site configurations in the upsampled dataset is smaller than in the standard bootstrap replicate dataset because of l ≪ L. Users need to ensure that sufficiently large little samples (*l* ≥ 10,000 sites) are utilized, as well as enough little samples (*s* ≥ 10) and bootstrap replicates (*r* ≥ 10) to generate reliable estimates. An ML tree is inferred from each little bootstrap replicate dataset, and the LBS support for a node/clade is the proportion of times that appeared in the little bootstrap replicate phylogenies ( $\hat{BCL}$ ). Each ML tree is dated using RelTime to generate node ages and CIs, and then, time estimates are summarized on the LBS consensus tree.

In the new approach (RelTime-JA), phylogenetic uncertainty is incorporated in the analysis by using the little bootstraps method framework and dating phylogenies produced in each little bootstrap replicate. Figure 2 shows an outline of this process for the little bootstraps method (Figure 2B), which is contrasted with the standard bootstrap method (Figure 2A). In the case of the standard bootstrap method, we generate bootstrap-resampled datasets (*A*_*i*_s), each obtained by randomly sampling sites with replacement from the original sequence alignment. *A*_*i*_ is subjected to ML phylogenetic analysis to infer the replicate phylogeny and branch lengths (*P*_*i*_). Then, the RelTime (Tamura et al., 2012) method is applied to *P*_*i*_, along with calibrations, to generate a replicate timetree containing divergence times and confidence intervals (*T*_*i*_). This process is applied to every *A*_*i*_ alignment. We choose RelTime for relaxed clock dating because its computational requirements are a small fraction of Bayesian methods (Tao et al., 2020). Moreover, RelTime has been reported to perform as well as Bayesian methods for dating phylogenies using empirical (Mello et al., 2017; Battistuzzi et al., 2018; Tao et al., 2020) and simulated data (Barba-Montoya et al., 2020; Mello et al., 2021). However, any other dating method can be used instead (Tao et al., 2020; Barba-Montoya et al., 2021).

Using the collection of bootstrap timetrees, we infer a consensus tree as outlined in the work of Felsenstein (1985). Then, we estimate the age for every inferred clade by mapping *T*_*i*_ timetrees onto the consensus tree clade by clade. For clade *j* in the bootstrap consensus tree, we first build a collection of member taxa and then find the most recent common ancestor (MRCA) of this set of taxa in every bootstrap replicate timetree. If *r* replicates have been conducted, we produce *r* age estimates for each node in the bootstrap consensus tree and their respective confidence intervals. The MRCA is used because the member taxa in the inferred clades in the consensus tree will not always be monophyletic in the replicate timetrees due to phylogenetic uncertainty. The mean of *r* age estimates (*t*_*j*_) is the age of clade *j* in the BS consensus phylogeny. The mean of the lower and upper bounds of the time estimates in the replicate timetrees establishes the confidence intervals for the age of clade *j* (*U*_*j*_, *L*_*j*_).

For datasets with long sequence alignments, the standard bootstrap approach is replaced by the bag of little bootstraps method (Figure 2B). In the little bootstrap approach, *r* bootstrap replicate alignments (*B*_*i*_) of *s* little subsamples of sites are analyzed. Using each *B*_*i*_, we first infer an ML tree (*PB*_*i*_) and subject it to relaxed clock dating to generate a replicate timetree (*TB*_*i*_). This procedure generates *r × s* timetrees. We then infer a little bootstrap consensus tree following the work of Sharma and Kumar (2021) and then map the time estimates from *TB*_*i*_ timetrees onto the consensus tree clade by clade as described previously for the standard bootstrap approach. The R codes developed for summarizing time estimates and constructing timetrees are available at https://github.com/josebarbamontoya/pu_dating.

### 2.2 RelTime-JA with little bootstraps for phylogenomic data

We used the RelTime-JA method with little bootstraps (Figure 2B) to analyze six phylogenomic datasets (Pessoa-Filho et al., 2017; Johnson et al., 2018; Ran et al., 2018; Sann et al., 2018; Kuntner et al., 2019; Álvarez-Carretero et al., 2022), which contained 89,212–33,173,174 nucleotide sites and 15–189 sequences (Supplementary Table S2). All phylogenomic datasets are available at https://doi.org/10.6084/m9.figshare.22114943. The outgroup clade of four datasets was pruned down to one species (Johnson et al., 2018; Ran et al., 2018; Sann et al., 2018; Kuntner et al., 2019). LBS replicates for each phylogenomic dataset were computed using LBS software. We ensured that the size of the little samples (*l*), the number of little samples (*s*), and bootstrap replicates (*r*) for each dataset were sufficient to compute a reliable consensus phylogeny and support values (Supplementary Table S2). For each bootstrap replicate, an ML phylogeny was inferred using the correct substitution model (Supplementary Table S2) in IQ-TREE (Nguyen et al., 2015). Each ML bootstrap tree was then individually dated using RelTime to generate node ages and CIs. Then, node times and CI bounds for each node were summarized using the dated bootstrap trees on the LBS consensus trees. We recommend using a large number of sites in the little samples (*l* ≥ 20,000 sites), as well as enough little samples (*s* ≥ 10) and bootstrap replicates (*r* ≥ 10), to generate reliable consensus phylogeny and LBS node support values.

For each analysis, we used a time constraint implemented as a narrow uniform distribution U (min, max) for the rooting ingroup node based on the time estimates from the original studies. We did not apply internal calibrations to assess the power of RelTime-SA and RelTime-JA in dealing with phylogenetic uncertainty (Supplementary Table S2). The rooting outgroup was excluded from the analysis. We evaluated the performance of RelTime-JA by comparing the node times and CIs with those from RelTime-SA. For each RelTime-SA, we used the ML phylogeny inferred in IQ-TREE using the substitution model from the original study. The same time constraints as for the RelTime-JA were used. We made an additional RelTime-JA with the little bootstraps method analysis of the apoid dataset (Sann et al., 2018) using 10 constraints (Supplementary Table S2). We evaluated the performance of RelTime-JA by comparing the MRCA node times with the RelTime-SA node time estimated using the same time constraints as for the RelTime-JA.

#### 2.2.1 Calculation of the computing time for phylogenomic data analysis

We evaluated the computing time required for the analysis of subsets of 1,000, 5,000, 10,000, 25,000, 50,000, and 100,000 sites from the concatenation alignment of 72 mammal sequences and 33,173,174 nucleotide sites from the study by Álvarez-Carretero et al. (2022). In BEAST2, we used the uncorrelated relaxed clock model. We used an autocorrelated clock model in MrBayes. The sequence likelihood was calculated under the HKY+Γ5 model, and a uniform tree prior was applied. The time unit was set at 100 Myr. The timetrees were computed applying one calibration on the ingroup node, which specified assigning a narrow uniform distribution U (139, 144 Ma), and one on the rooting outgroup node specified assigning U (200, 205 Ma) based on the time estimates from the original study. We calculated the computing time for each analysis to reach a minimum effective sample size (ESS) of 200 for all the parameters using only one thread. By extrapolating these results to 33,173,174 sites, we estimated the expected computing time required to reach a minimum ESS of 200 for all parameters. We also compared the expected Bayesian computing times with the computing time required by the RelTime-JA using the little bootstraps method for analyzing the dataset used by Álvarez-Carretero et al. (2022).

### 2.3 Molecular clock dating analysis of simulated data

#### 2.3.1 Computer-simulated data

We used datasets previously simulated by Tamura et al. (2012). The model timetree consisted of 446 species derived from the bony-vertebrate clade in the Timetree of Life (Hedges and Kumar 2009), from which we randomly sampled 51 taxa. We chose nucleotide gene alignments in which the rate variation was autocorrelated such that the rate of a descendant branch was drawn from a lognormal distribution around the mean rate of the ancestral branch. An autocorrelation parameter *ν* =1 was used (Kishino et al., 2001). All datasets were generated using SeqGen (Rambaut and Grassly, 1997) under the Hasegawa–Kishino–Yano (HKY) substitution model (Hasegawa et al., 1985) and heterogeneous sets of evolutionary parameters, including sequence lengths (258–9,353 sites), evolutionary rates (range 1.35–2.60 substitutions per site per billion years), G+C-content bias (G+C contents range 39%–82%), and transition/transversion rate bias (transition/transversion ratio, range 1.9–6.0). More details are given in the study by Tamura et al. (2012). We used 11 alignments of 309, 450, 537, 782, 1,073, 1,523, 2,116, 3,100, 4,070, 7,002, and 9,359 sites each, with different levels of topological errors—normalized Robinson–Foulds (RF) distance (Robinson and Foulds, 1981) of 0.21, 0.27, 0.08, 0.21, 0.13, 0.04, 0.10, 0.06, 0.00, 0.13, and 0.04, respectively. The normalized RF distances were calculated using the R function *MultiRF* (Revell, 2012) by comparing the model timetree with the ML trees inferred in MEGA-CC for macOS (Stecher et al., 2020), applying the HKY+Γ5 model (Hasegawa et al., 1985; Yang, 1994). The simulated datasets and model timetree are available at https://doi.org/10.6084/m9.figshare.22114943.

#### 2.3.2 BEAST2 analysis

We analyzed 11 datasets using BEAST2 (Bouckaert et al., 2019) under the *Relaxed Clock Log Normal* (ucld) model, which assumes that the substitution rates for branches are independent variables from a lognormal distribution (Drummond et al., 2006). The lognormal distribution is parametrized using the mean and the standard deviation. The mean (*ucldMean.c*) was assigned a gamma hyperprior G (2, 0.1) with a mean of 0.2, and the standard deviation (*ucldStdev.c*) was assigned a gamma hyperprior G (2, 0.05) with a mean of 0.1. The sequence likelihood was calculated under the HKY+Γ5 model. The time unit was set at 100 Myr. For the tree prior, hyperpriors were assigned to the parameters in the birth–death-sampling model, the net diversification rate $\lambda -\mu ∼U\left(0,1\right)$ , and the relative extinction rate $\mu /\lambda ∼U\left(0,1\right)$ (Stadler, 2010; Höhna et al., 2011). The timetrees were computed applying one calibration on the ingroup node, specified assigning a uniform distribution U (445, 465 Ma) and a correct age constraint U (526, 527 Ma) on the rooting outgroup node to ensure that the height of the inferred timetrees matches the height of the model tree. Analyses were performed by either fixing the inferred ML tree topology (BEAST2-SA) or jointly inferring the topology and divergence times to build a timetree (BEAST2-JA). No internal calibrations were applied. We ran each analysis four times to ensure convergence and that ESS values were all >100. We then merged the samples from the runs before summarizing the posterior. Each run consisted of 1×10^8^ iterations, sampling every 5,000. The burn-in was set to 10% of samples, resulting in a total of 7.2×10^4^ samples from all four runs.

#### 2.3.3 MrBayes analysis

In MrBayes (Ronquist, et al., 2012b), we used the autocorrelated lognormal model (TK02) where the rates for branches are autocorrelated variables from a lognormal distribution (Thorne and Kishino, 2002). In the TK02 model, a single parameter (*tk02varpr*) controls the rate variation across the tree. The mean is assigned a lognormal hyperprior LN (−0.155, 0.2), with the mean exp{−0.155,0.2^2^/2} = 0.2. The variance (*tk02varpr*) was assigned an exponential hyperprior with a mean of 0.1. The sequence likelihood was calculated under the HKY+Γ5 model. The time unit was set at 100 Myr. A uniform tree prior was used. The timetrees were computed applying one calibration on the ingroup node, specified assigning a uniform distribution U (445, 465 Ma) and a correct age constraint U (526, 527 Ma) on the rooting outgroup node to ensure that the height of the inferred timetrees matches the height of the model tree. Analyses were performed by either fixing the inferred ML topology (MrBayes-SA) or by jointly inferring the topology and divergence times to build a timetree (MrBayes-JA). No internal calibrations were applied. We ran each analysis eight times to ensure convergence and that ESS values were all >100. We then merged the samples from the runs before summarizing the posterior. Each run consisted of 1×10^7^ iterations, sampling every 500, with the burn-in set to 10% of samples, resulting in a total of 1.44×10^5^ samples from the eight runs.

#### 2.3.4 RelTime analysis

For the RelTime-SA, we used RelTime implemented in MEGA-CC for macOS (Stecher et al., 2020). They were prototyped in MEGA X (Kumar et al., 2018). We used the inferred ML phylogeny with branch lengths for each dataset analysis to infer node times and CIs. Timetrees were computed by applying one calibration on the ingroup node which was assigned a uniform distribution U (445, 465 Ma). Using no internal calibrations allowed us to directly assess the power of RelTime-SA and RelTime-JA in dealing with phylogenetic uncertainty. Dates for all taxa in the outgroup were excluded because RelTime analysis does not produce estimates in the outgroup (for an explanation, refer to the work of Tamura et al., 2012; 2018). For the RelTime-JA, we developed a new method based on a bootstrap (BS) resampling approach (Felsenstein, 1985) for inferring timetrees with phylogenetic uncertainty (Figure 2A). LBS software computed 100 bootstrap replicates for each simulated sequence alignment (Sharma and Kumar, 2021) to generate reliable consensus phylogeny and bootstrap node support values. An ML phylogeny was inferred using the HKY+Γ5 substitution model in MEGA-CC for each bootstrap replicate. Each ML bootstrap tree was then individually dated using RelTime to generate node ages and CIs under the same parameters used for the RelTime-SA. Then, node times and CI bounds for each node were summarized using 100 dated bootstrap trees on the BS consensus tree. The rooting outgroup was excluded from the analysis. The final timetrees include BS support values for the clades.

## 3 Results

### 3.1 Divergence time estimation of phylogenomic data by applying the RelTime-JA with little bootstraps method

We analyzed a phylogenomic dataset of 177 species of apoids (wasps + bees) and 283,008 nucleotide sites (Sann et al., 2018). We built a timetree using RelTime-SA and compared it with RelTime-JA with the little bootstraps method (Figure 2B). This dataset was selected because despite a very large number of sites, many clade relationships received less than 80% bootstrap support. Sann et al. (2018) used 10 calibrations in their Bayesian-SA (Supplementary Table S2). We used their alignment and calibrations to test if RelTime-SA and RelTime-JA produce similar estimates. We ensured that sufficiently long little samples and a sufficiently large number of subsamples and bootstrap replicates were utilized in the LBS analysis (Supplementary Table S2). The average LBS node support was 0.96, with several node support values lower than 0.7, similar to the BS support values in the inferred phylogeny from the original study.

**FIGURE 3:**
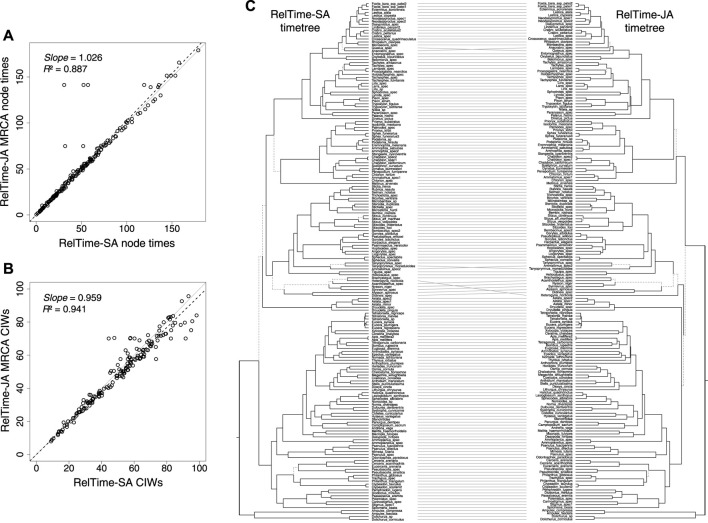
**(A)** Comparison of the original MCMTree time estimates and time estimates obtained by using RelTime-JA with little bootstraps for an apoid (wasps + bees) phylogenomic dataset of 177 species and 283,008 sites from the study by Sann et al. (2018). **(B)** Comparison of the original NCIWs (CI width/true time × 100) and RelTime-JA NCIWs. The *slope* and coefficient of determination (*R* ^2^) for the linear regression through the origin are shown. The black dashed lines represent the best-fit linear regression through the origin. The solid gray line represents equality between estimates. **(C)** Tanglegram comparing timetrees obtained by Sann et al. (2018) and RelTime-JA timetrees. Dotted line edges represent distinct edges between the timetrees.

RelTime-SA and RelTime-JA produced similar estimates for a vast majority of node times and CIs, but five MRCA node ages were considerably older and had wider CIs in RelTime-JA. They stand out as outliers in Figures 3A, B. These differences occurred because of large topological rearrangements between SA and JA topologies, where the positions of *Heterogyna nocticola, Astata* and *Dryudella* species, and some other clades shifted significantly (Figure 3C). Other differences were observed for relationships near the tips of the tree where the evolutionary change was small. We also observed that RelTime-SA generated wider CIs than RelTime-JA for several deep nodes in the timetree (Figure 3B). This difference in estimated times suggests that phylogenetic uncertainty can considerably impact time estimates.

**FIGURE 4:**
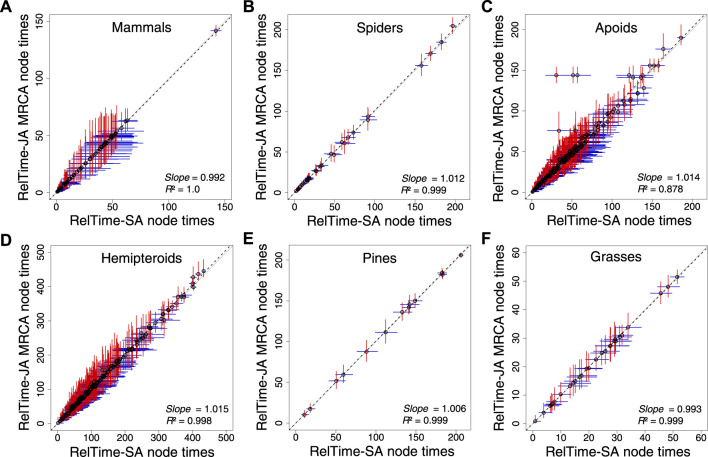
Comparison of time estimates obtained by using RelTime-SA and RelTime-JA with little bootstraps for six phylogenomic datasets: **(A)** mammals, **(B)** spiders, **(C)** apoids, **(D)** hemipteroids, **(D)** pines, and **(F)** grasses. CIs are represented for RelTime-SA (blue lines) and RelTime-JA (red lines). For RelTime-JA, we used the estimated node times for the MRCA of all the sets of taxa in the original phylogenies. The *slope* and coefficient of determination (*R* ^2^) for the linear regression through the origin are shown. The black dotted line represents the best-fit linear regression through the origin. The solid gray line represents equality between estimates.

We analyzed the apoid and five other phylogenomic datasets (Pessoa-Filho et al., 2017; Johnson et al., 2018; Ran et al., 2018; Sann et al., 2018; Kuntner et al., 2019; Álvarez-Carretero et al., 2022) applying only a root calibration, which is critical to directly assess the impact of applying SA or JA methods on time estimates without topological and time constraints. RelTime-JA generated very similar node times and CIs to RelTime-SA (Figure 4) because phylogenetic uncertainty was small for the analyzed phylogenomic datasets. However, some nodes in the hemipteroid and spider timetrees differed considerably. On average, RelTime-JA generated 5% wider CIs than RelTime-SA, which suggested a small effect of phylogeny uncertainty. RelTime-JA with little bootstraps generated timetree topologies that differed by less than 7% from the published timetrees, with much of the difference observed near the tips of the phylogeny (Supplementary Table S2). The average little bootstraps support value for RelTime-JA timetrees was >95% for all datasets (Supplementary Table S2).

### 3.2 Impact of phylogenetic uncertainty on Bayesian and RelTime time estimates

**FIGURE 5:**
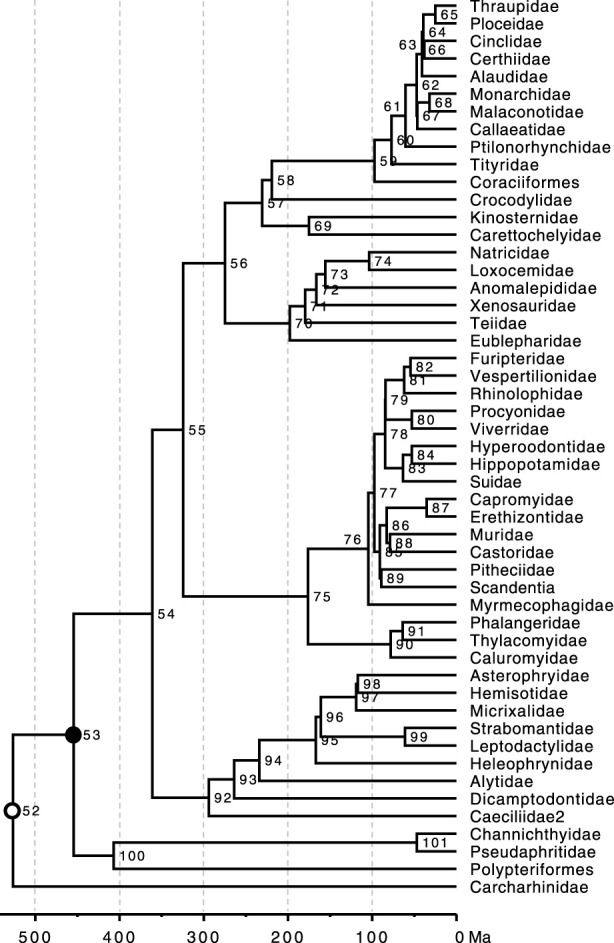
Phylogeny of 51 taxa showing calibrated nodes. The tree has been scaled to time based on TEs from the Timetree of Life (Hedges and Kumar, 2009). Calibrations are represented for two nodes. 1) A uniform distribution U (min, max) for the rooting ingroup calibration U (444.6, 464.6 Ma) was applied in BEAST2, MrBayes, and RelTime analyses (closed black dot). 2) In BEAST2 and MrBayes analyses, a root constraint was (open black dot) implemented as a uniform distribution U (526, 527 Ma).

**FIGURE 6:**
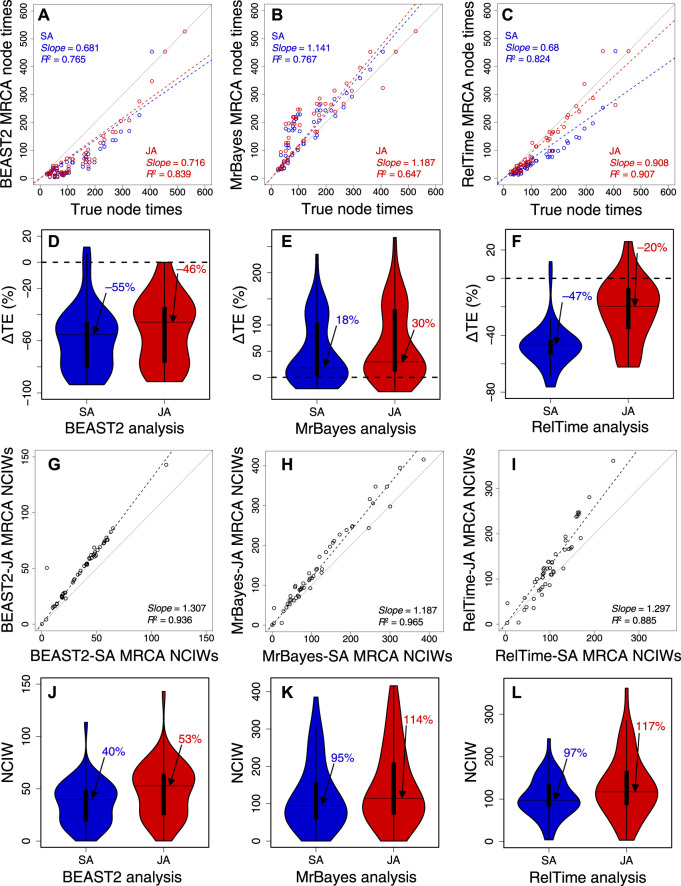
Comparison of time estimates obtained by using SA (blue dots) and JA (red dots) methods with true node times for **(A)** BEAST2, **(B)** MrBayes, and **(C)** RelTime for a simulated dataset of 450 sites. The *slope* and coefficient of determination (*R* ^2^) for the linear regression through the origin are shown. The blue (SA) and red (JA) dashed lines represent the best-fit linear regression through the origin. The solid gray line represents equality between estimates. Distributions of the differences between the estimated and true node times (ΔTEs) for times inferred by SA (blue) and JA (red) in **(D)** BEAST2, **(E)** MrBayes, and **(F)** RelTime. The black horizontal line represents the median value, indicated by an arrow. Comparison of NCIWs (CI width/true time × 100) obtained by using SA and JA methods with true node times for **(G)** BEAST2, **(H)** MrBayes, and **(I)** RelTime. The *slope* and coefficient of determination (*R* ^2^) for the linear regression through the origin are shown. The black dashed lines represent the best-fit linear regression through the origin. The solid gray line represents equality between estimates. Distribution of NCIWs of all the nodes for times inferred by SA (blue) and JA (red) for **(J)** BEAST2, **(K)** MrBayes, and **(L)** RelTime. The black horizontal line represents the median value, indicated by an arrow. For both SA and JA methods, we used the estimated node times for the MRCA of all the sets of taxa in the model timetree.

Bayesian-JA is computationally expensive for large phylogenomic datasets, and the number of phylogeny errors is usually small. So, we evaluated the difference between JA and SA estimates for a rather short sequence dataset (450 nucleotide sites). We chose a simulated dataset because it allowed us to choose a situation where the ML phylogeny contained many errors. We scanned datasets simulated by Tamura et al. (2012) and chose a sequence alignment in which 27% of the inferred clades in the ML tree were incorrect. The ML tree inferred from this dataset had errors on the terminal, intermediate, and deep nodes because the sequence length of the simulated gene was relatively short. We compared JA and SA time estimates produced by applying BEAST2 (Bouckaert et al., 2019), MrBayes (Ronquist et al., 2012b), and RelTime (Figure 6). For RelTime-JA, we used the standard BS method (Figure 2A). In this comparison, the true ages of clades in the true tree (Figure 5) were compared with the MRCA of member taxa in the correct clades in the trees produced by using SA and JA methods.

BEAST2 produced underestimates of node times (Figure 6A), but the *slope* and *R* ^2^ were marginally better in JA (*slope* = 0.72; *R* ^2^ = 0.84) than in SA (*slope* = 0.68; *R* ^2^ = 0.77). MrBayes produced overestimates (Figure 6B), but the *slope* and *R* ^2^ were slightly better for SA (*slope* = 1.14; *R* ^2^ = 0.77) than those for JA (*slope* = 1.19; *R* ^2^ = 0.65). Therefore, SA and JA produced comparable node times for BEAST2 and MrBayes. In contrast, the *slope* and *R* ^2^ for RelTime-JA (*slope* = 0.91; *R* ^2^ = 0.91) were considerably better than those for RelTime-SA (*slope* = 0.68, *R* ^2^ = 0.82) (Figure 6C). So, JA performed better than SA for this example dataset in RelTime.

We also quantified the accuracy of the SA and JA methods by computing the difference between the estimated MRCA node times and the true node times. The difference was divided by the true node time and multiplied by 100 to generate a percent time error (ΔTE). In BEAST2, many node times were underestimated, resulting in an overall tendency to underestimate times. The distribution of ΔTE and its median for BEAST2-SA exhibited greater underestimation than those for BEAST2-JA, with a median ΔTE of −55% compared to −46% (Figure 6D). The median ΔTE from MrBayes-SA and MrBayes-JA was also significantly different, 18% and 30%, respectively (Figure 6E). The median ΔTE for RelTime-SA was larger than that for RelTime-JA (−47% vs.–20%; Figure 6F). These differences in time estimates between SA and JA methods for the same method show that phylogenetic uncertainty can significantly impact the bias and accuracy of time estimates.

We measured the accuracy of CIs by coverage probability (CP), which is the proportion of nodes containing the true node times in the CIs generated by the given method. We also calculated normalized CI widths (CI width/true time × 100; NCIW) for node times. In BEAST2-SA, the CP (0.06) was significantly lower than that for BEAST2-JA (0.28). CIs (Figure 6G) were considerably wider for BEAST2-JA than for BEAST2-SA (median NCIW of 53% and 40%, respectively; Figure 6J). MrBayes-JA also generated wider CIs than MrBayes-SA (Figure 6H). Although the median NCIW for MrBayes-JA (114%) was significantly wider than for MrBayes-SA (95%), the CP was higher for MrBayes-SA (0.76) than for MrBayes-JA (0.70) due to a smaller overestimation of time estimates for MrBayes-SA (Figure 6K). RelTime-JA CIs were also considerably wider than in RelTime-SA (Figure 6I), the median NCIW for RelTime-JA was 117% compared to 97% for RelTime-SA (Figure 6L), and thus, the CP for RelTime-SA (0.71) was significantly lower than that for RelTime-JA (0.92). The wider CIs estimated using the JA method show that phylogenetic uncertainty significantly impacts CIs, suggesting that using the JA method in BEAST2, MrBayes, and RelTime will be needed to incorporate phylogenetic uncertainty in the CIs.

**FIGURE 7:**
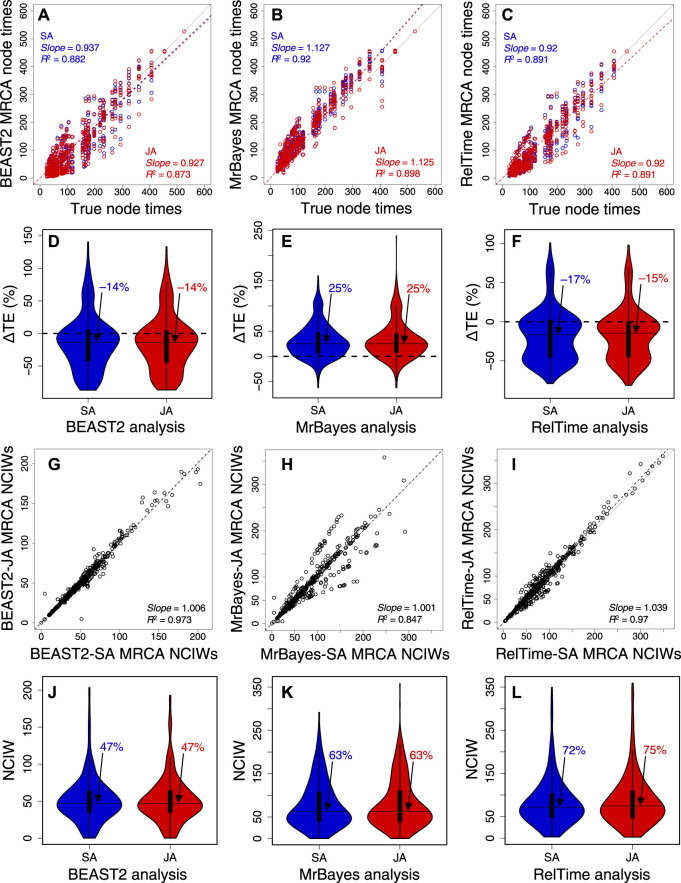
Comparison of composite time estimates across 10 simulated dataset analyses obtained by using SA (blue dots) and JA (red dots) methods with true node times for **(A)** BEAST2, **(B)** MrBayes, and **(C)** RelTime for a simulated dataset of 450 sites. The *slope* and coefficient of determination (*R* ^2^) for the linear regression through the origin are shown. The blue (SA) and red (JA) dashed lines represent the best-fit linear regression through the origin. The solid gray line represents equality between estimates. Distributions of the differences between the estimated and true node times (ΔTEs) for times inferred by SA (blue) and JA (red) in **(D)** BEAST 2, **(E)** MrBayes, and **(F)** RelTime. The black horizontal line represents the median value, indicated by an arrow. Comparison of NCIWs (CI width/true time × 100) obtained by using SA and JA methods with true node times for **(G)** BEAST2, **(H)** MrBayes, and **(I)** RelTime. The *slope* and coefficient of determination (*R* ^2^) for the linear regression through the origin are shown. The black dashed lines represent the best-fit linear regression through the origin. The solid gray line represents equality between estimates. Distribution of NCIWs of all the nodes for times inferred by SA (blue) and JA (red) for **(J)** BEAST2, **(K)** MrBayes, and **(L)** RelTime. The black horizontal line represents the median value, indicated by an arrow. For both SA and JA methods, we used the estimated node times for the MRCA of all the sets of taxa in the model timetree.

We further analyzed 10 other datasets of 309, 537, 782, 1,073, 1,523, 2,116, 3,100, 4,070, 7,002, and 9,359 sites (Supplementary Table S3) to evaluate the generality of the aforementioned trends (Figure 7). In BEAST2, MrBayes, and RelTime, the *slope* and *R* ^2^ of node time estimates from SA and JA methods were similar (Figures 7A–C, Supplementary Figures S1, S3, S5). This made the distribution and median ΔTEs from SA and JA similar (Figures 7D–F, Supplementary Figures S1A, S3A, S5A). CPs were consistently similar between SA and JA methods (Supplementary Figures S2A, S4A, S6A), which means that JA did little to improve the results from SA. The relative NCIWs did not show considerable differences between SA and JA (Figure 7G–L) across all datasets. However, some datasets displayed larger differences (Supplementary Figures S2B, S4B, S6B).

**FIGURE 8:**
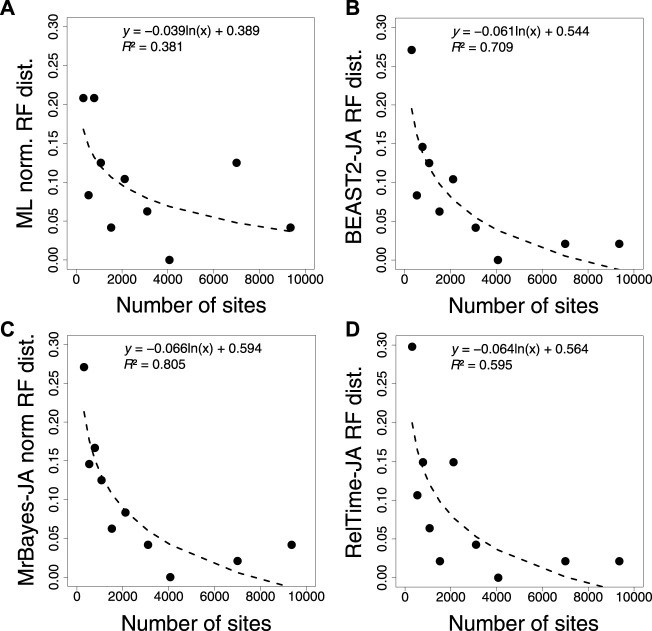
Correlation between the number of sites and normalized RF distance for **(A)** ML, **(B)** BEAST2, **(C)** MrBayes, and **(D)** RelTime-JA with standard bootstrap phylogenies. The equation and coefficient of determination (*R* ^2^) for the logarithmic regression are shown. The black dashed line represents the best-fit logarithmic regression.

Furthermore, we compared the normalized RF distance from each inferred tree using ML, BEAST2, MrBayes, and RelTime-JA with the standard bootstrap method (Figures 8A–D). We found that applying the JA method did not increase the accuracy of phylogeny inference (Supplementary Table S3). The timetrees inferred by applying the JA method implemented in BEAST2, MrBayes, and RelTime-JA generated RF distances similar to those of the ML tree. This is reasonable because the divergence time estimation for an individual bootstrap replicate does not add any new phylogenetic information, as is the case for the Bayesian posterior trees. Therefore, SA and JA do not produce very different results even when the inferred phylogenies contain many errors, except in extreme cases.

## 4 Discussion

Phylogenomic datasets now contain many taxa and sites. The large number of taxa increases the phylogenetic uncertainty, and very long sequences can increase the time required for analysis. We show that the little bootstraps can generate a collection of phylogenies, followed by relaxed clock dating to capture the phylogenetic uncertainty, similar to molecular dating using Bayesian approaches. In this approach, the bag of little bootstraps analysis can infer molecular timetrees that incorporate phylogenetic uncertainty and alleviate the computational burden caused by long sequences simultaneously. We observed good performance of our new method to estimate divergence times even without using calibrations, which allowed us to examine our method’s power in dealing with phylogenetic uncertainty.

We found that the topological accuracy of the inferred timetrees was very similar between SA and JA methods for Bayesian and RelTime approaches. However, in the cases with significant variations among tree topologies, such discrepancies resulted in differences in estimated times. This can be attributed to the specific method employed to infer the timetrees (SA or JA). We found that topological shifts on internal, deep, and long branches and substantial lineage rearrangements can generate a considerable difference in time estimates between SA and JA methods. Therefore, we suggest assessing the impact of phylogenetic uncertainty on time estimates by comparing the timetree node time estimates from SA and JA methods.

We show that our RelTime-JA with the little bootstraps method generates reliable timetree topologies and time estimates. Furthermore, it considerably decreases the computation time for JA time estimation. In phylogenomics, these savings can be substantial and remain low as the sequence alignment length increases from thousands to millions of sites. We calculated the computing time for RelTime-JA with the little bootstraps method required to analyze the whole dataset used by Álvarez-Carretero et al. (2022). We estimated a 614× time saving compared to the required computing time for BEAST2 and a 704× time saving compared to MrBayes. These analyses could be parallelized, but for the Bayesian methods, this would still require a long computing time per MCMC chain.

Although the primary focus of this article is to compare JA and SA for the same software, it is crucial to acknowledge that different software applications generated notably distinct time estimates. Notably, we observed significant variations in estimates between Bayesian software due to the different specifications of relaxed clock models, such as independent rates in BEAST2 and autocorrelated rates in MrBayes.

While the focus of this article is primarily on comparing JA and SA for the same software application, it is important to note that different software applications generated different time estimates. In some cases, we observed a significant difference in estimates between Bayesian software, which was partly caused by the different specifications of the relaxed clock model, such as independent rates in BEAST2 and autocorrelated rates in MrBayes. Additionally, with short sequences, the tree prior tends to have considerably more influence (May et al., 2021). For BEAST2, we specified the birth–death tree prior, while for MrBayes, we used a uniform tree prior. It has been reported that the uniform tree prior implemented in MrBayes can be strongly informative in terms of divergence time estimation (May et al., 2021). In RelTime, it is not required to specify a tree prior or clock model for evolutionary rates to account for the heterogeneity of branch rates. Instead, RelTime directly calculates relative times and lineage rates based on the inferred branch lengths obtained from molecular sequences. We found that RelTime time estimates fell between BEAST2 and MrBayes estimates for both SA and JA methods. Moreover, we found that RelTime-JA generated node support values comparable with Bayesian posterior node probabilities in BEAST2, with lower values for timetrees inferred with higher topological error from shorter alignments and higher values for timetrees inferred with lower topological error from longer alignments. MrBayes, however, generated much higher node posterior probabilities in all cases, even for datasets with high topological error (Supplementary Table S3).

Overall, the results presented here demonstrate that phylogenetic uncertainty can impact time estimates considerably for some nodes in the phylogeny, particularly for datasets with short sequences. This prompts us to use the JA approach that deals with phylogenetic uncertainty. Ultimately, the complexities of how evolution proceeds, and whether this is effectively described by current dating methods, will determine whether the phylogenetic uncertainty impacts time estimates or not.

## Data Availability

Publicly available datasets were analyzed in this study. The datasets underlying this article are available in the figshare repository at https://doi.org/10.6084/m9.figshare.22114943. The R codes for summarizing time estimates and constructing timetrees are available at https://github.com/josebarbamontoya/pu_dating.
