# Supplementary material for: Molecular timetrees using relaxed clocks and uncertain phylogenies
Source: Front Bioinform. 2023 Aug 3;3:1225807. doi: 10.3389/fbinf.2023.1225807 (PMC10435864; doi:10.3389/fbinf.2023.1225807)
Supplement: Supplementary file 1 [file DataSheet1.PDF]

Supplementary Material

Molecular timetrees using relaxed clocks and uncertain phylogenies

Jose Barba-Montoya, Sudip Sharma, Sudhir Kumar*

*** Correspondence:** Corresponding Author: s.kumar@temple.edu

List of Contents

Supplementary Figures S1-S6 (pages 2-7).

Supplementary Tables S1-S3 (pages 8-10).

References (page 11).

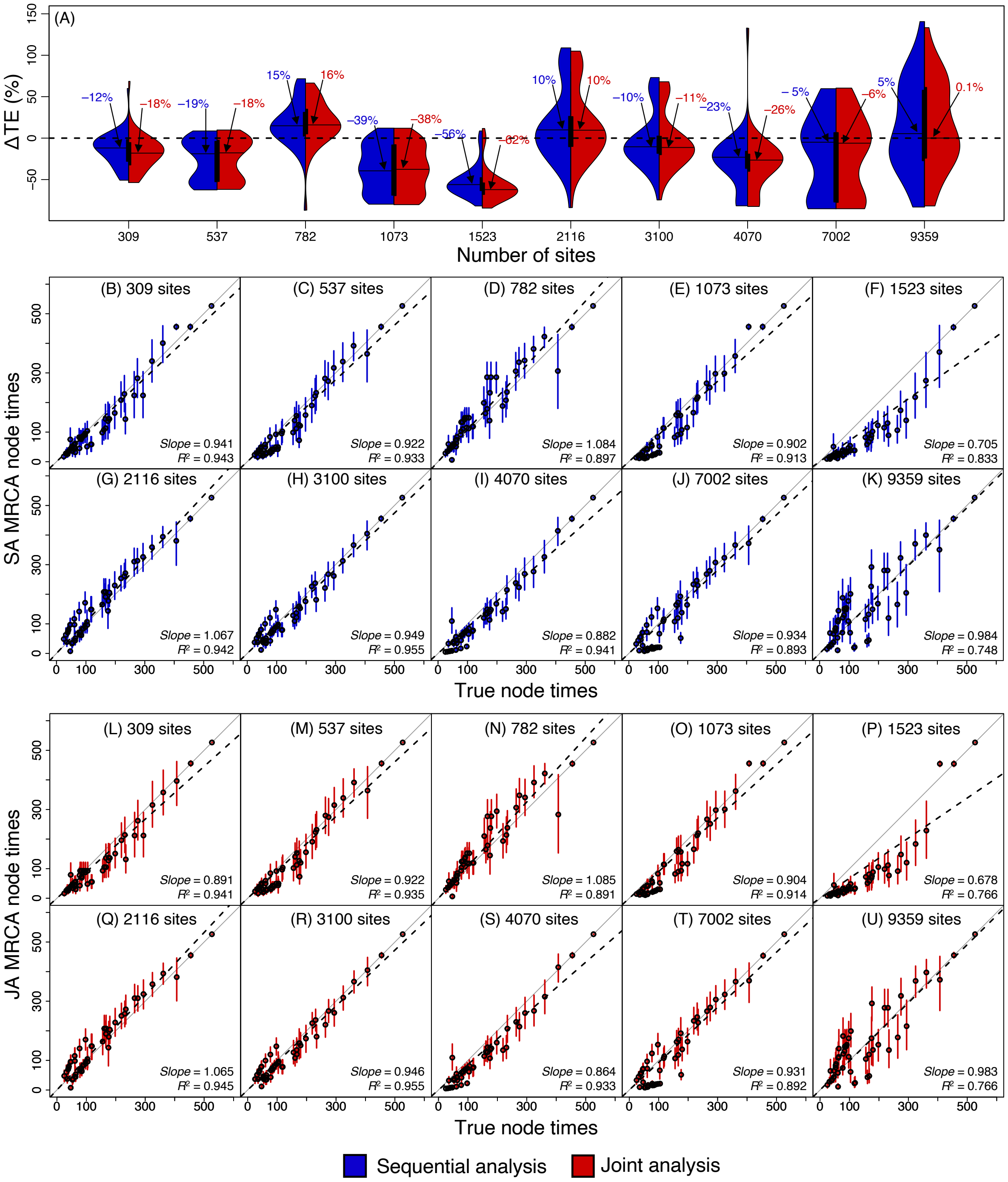

Supplementary Figure S1. (A) Distributions of the differences between estimated and true node times (ΔTEs) for times inferred by SA and JA methods in BEAST2. The black horizontal lines represent median values. (B-U) Comparison of time estimates obtained by using SA and JA in BEAST2 with true node times for 10 simulated datasets. 95% credibility intervals are represented for SA (blue lines) and JA (red lines). The *slope* and coefficient of determination (R^2) for the linear regression through the origin are shown. The black dotted line represents the best-fit linear regression through the origin. The solid gray line represents equality between estimates. For both SA and JA methods we used the estimated node times for the MRCA of all the sets of taxa in the model timetree.

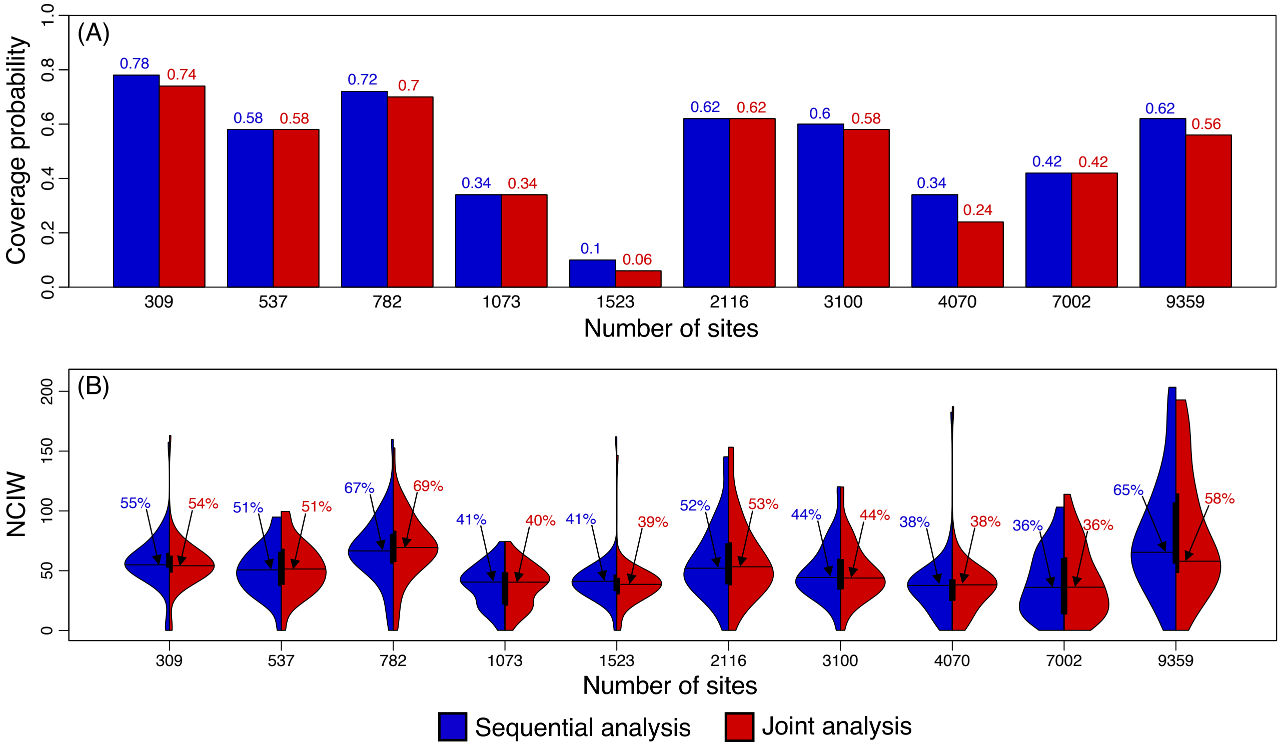

Supplementary Figure S2. (A) Coverage probability of CIs and (B) distribution of NCIWs (CI width/true time × 100) of all the nodes for ten simulated datasets. The performances of the SA (blue) and JA (red) methods implemented in BEAST2 are compared. The black horizontal lines in (B) represent median values. For both SA and JA methods we used the estimated node times for the MRCA of all the sets of taxa in the model timetree.

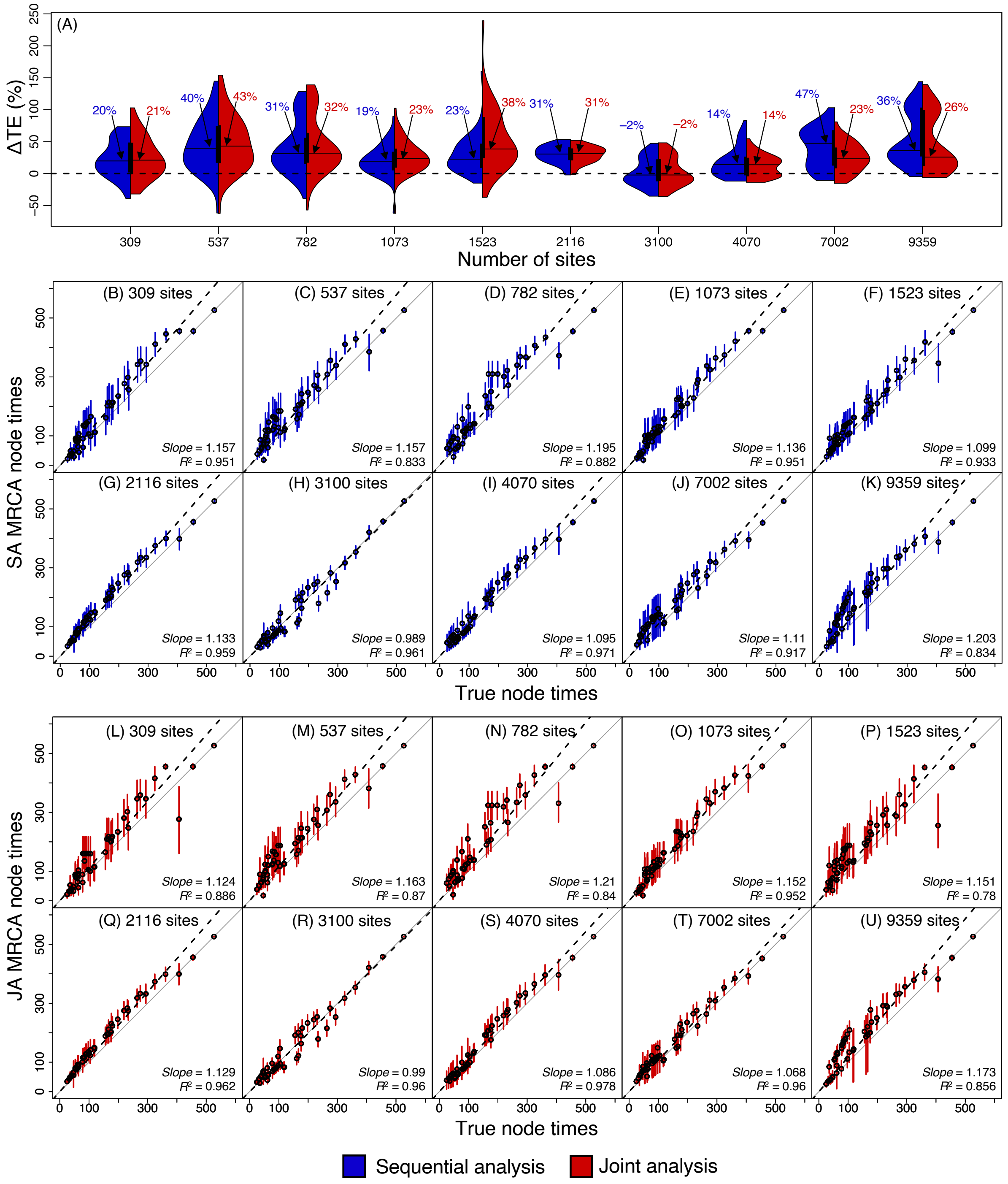

Supplementary Figure S3. (A) Distributions of the differences between estimated and true node times (ΔTEs) for times inferred by SA and JA in MrBayes. The black horizontal lines represent median values. (B-U) Comparison of time estimates obtained by using SA and JA in MrBayes with true node times for 10 simulated datasets. 95% credibility intervals are represented for SA (blue lines) and JA (red lines). The *slope* and coefficient of determination (R^2) for the linear regression through the origin are shown. The black dotted line represents the best-fit linear regression through the origin. The solid gray line represents equality between estimates. For both SA and JA methods we used the estimated node times for the MRCA of all the sets of taxa in the model timetree.

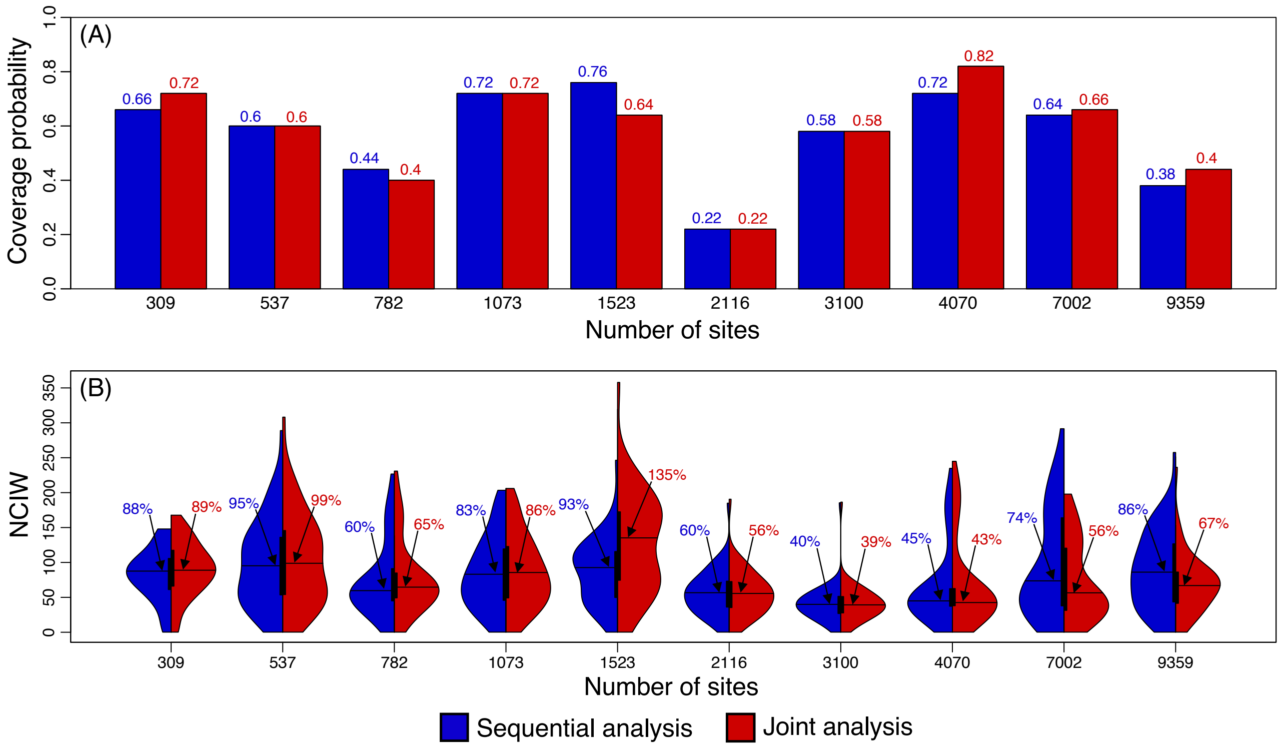

Supplementary Figure S4. (A) Coverage probability of CIs and (B) distribution of NCIWs (CI width/true time × 100) of al the nodes for ten simulated datasets. The performances of the SA (blue) and JA (red) methods implemented in MrBayes are compared. The black horizontal lines in (B) represent median values. For both SA and JA methods we used the estimated node times for the MRCA of all the sets of taxa in the model timetree.

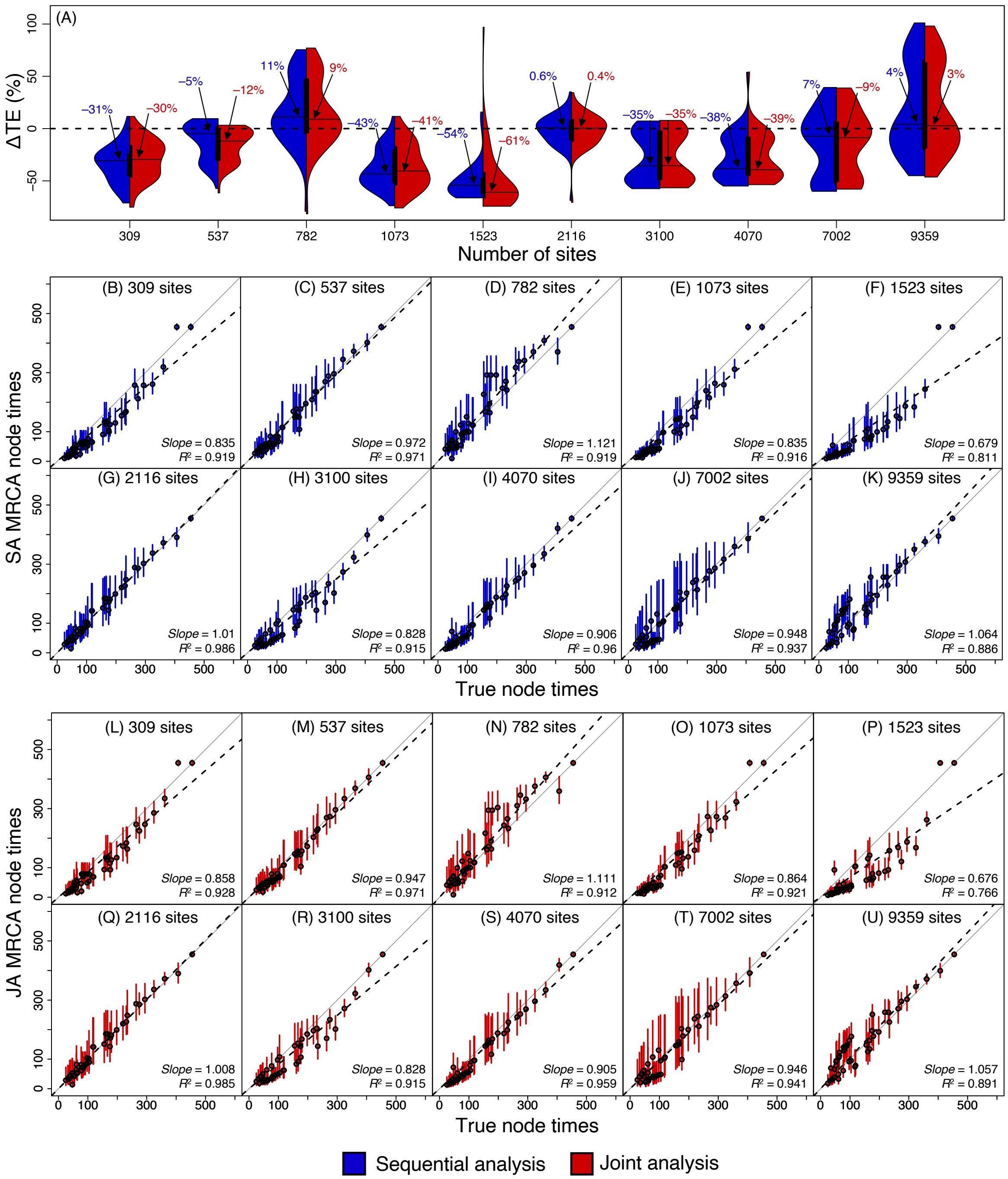

Supplementary Figure S5. (A) Distributions of the differences between estimated and true node times (ΔTEs) for times inferred by RelTime-SA and RelTime-JA with standard bootstrap methods. The black horizontal lines represent median values. (B-U) Comparison of time estimates obtained by using RelTime-SA and RelTime-JA with true node times for 10 simulated datasets. CIs are represented for SA (blue lines) and JA (red lines). The *slope* and coefficient of determination (R^2) for the linear regression through the origin are shown. The black dotted line represents the best-fit linear regression through the origin. The solid gray line represents equality between estimates. For both SA and JA methods we used the estimated node times for the MRCA of all the sets of taxa in the model timetree.

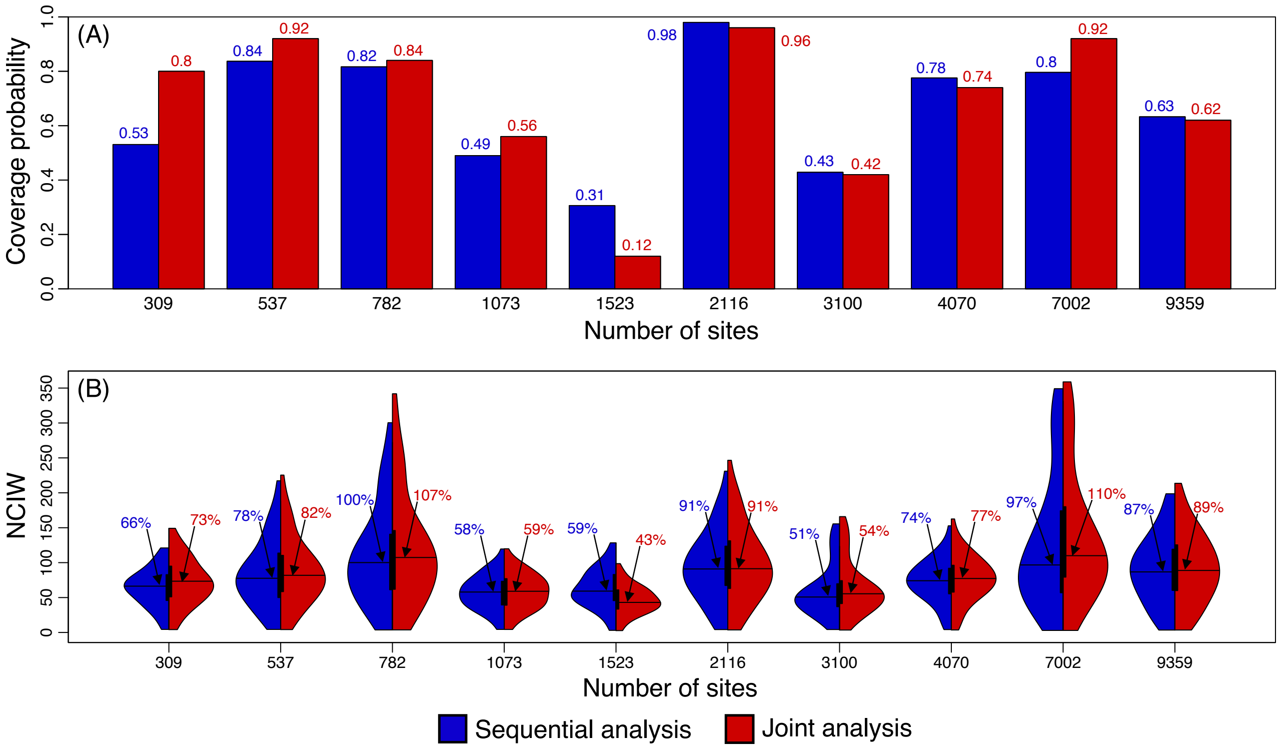

Supplementary Figure S6. (A) Coverage probability of CIs and (B) distribution of NCIWs (CI width/true time × 100) of al the nodes for ten simulated datasets. The performances of the RelTime-SA (blue) and RelTime-JA with standard bootstrap (red) methods are compared. The black horizontal lines in (B) represent median values. For both SA and JA methods we used the estimated node times for the MRCA of all the sets of taxa in the model timetree.

Supplementary Table S1. Comparison of computing time for phylogenomic data analysis.

Bayesian computing time (days)		
sites	BEAST2	MrBayes
1,000	1.14	7.0
5,000	6.2	13.1
10,000	11.94	18.1
25,000	20.04	33.7
50,000	35.78	47.53
100,000	56.04	69.95
33,173,174	~1,7914 (49 years)	~2,0544 (56 years)
RelTime-JA with little bootstraps computing time (days)		
100,000 subsample sites (s=10, r =10, g=0.665)	29.17	

CPU time required for the analysis of subsets from a concatenation alignment form Álvarez-Carretero et al. (2022) study. All analyses were computed using one thread.

Supplementary Table S2. Summary of phylogenomic data analysis by applying the RelTime-JA with little bootstraps method.

Dataset	No. of sites, *L*	No. of sequences, *S*	Distinct sites, *C*	Power factor, *g*	*s* × *r*	Sites, *l*	Substitution model	Time constraint	Norm RF distance	LBS mean node support	Refs*
Mammals	33,173,174	72	10,381,608	0.665	10×10	100,000	HKY+Γ5	U(1.37,1.47)	0.01	1.00	1
Pines	4,246,454	15	139,455	0.649	20×20	20,000	GTR+Γ5	U(2.01,2.11)	0.00	1.00	2
Apoids	283,008	177	210,220	0.789	10×10	20,000	GTR+Γ5	U(1.81,1.91)	0.07	0.96	3
Apoids (cal.)	283,008	177	210,220	0.789	10×10	20,000	GTR+Γ5	10 constraints	0.07	0.96	3
Grasses	135,243	30	23,848	0.78	20×20	10,000	GTR+Γ5	U(0.49,0.54)	0.00	0.99	4
Hemipteroids	131,013	189	100,508	0.84	10×10	20,000	GTR+Γ5	U(4.28,4.38)	0.03	0.96	5
Spiders	89,212	32	41,288	0.808	20×20	10,000	HKY+Γ5	U(1.92,2.02)	0.00	0.96	6

L and *S* are the total number of sites and sequences, respectively, in the full dataset. *C* is the number of unique site configurations in the full sequence alignment. The power factor (*g*), the number of little samples (*s*) and the number of replicates per little sample (*r*) were selected using the automatic procedure. We used a time constraint implemented as uniform distribution U(min, max) for the rooting ingroup node. Time unit was set at 100 Myr. For the Apoids (cal.) analysis, we used 10 constraints derived from the original calibrations, specifying a list of taxa whose MRCA is the node to calibrate: C1, split *Ampulex compressa – Foxita bara ssp patei1*, U(98.2,179). C2, split *Chlorion hirtum – Sceliphron curvatum*, min = 33.9. C3, *split Lindenius panzeri2 – Crabro peltarius*, min = 13.65. C4, split *Trypoxylon lactitarse – Pison atrum*, min = 13.65. C5, split *Sphecius convallis – Hoplisoides spec*, min = 33.9. C6, split *Stigmus spec1 – Spilomena beata*, min = 89.3. C7, split *Polemistus spec – Passaloecus eremita*, min = 34. C8, split *Ammoplanus spec – Melitta haemorrhoidalis*, min = 98.2. C9, split *Bombus rupestris – Tetragonula carbonaria*, min = 50. C10, split *Epeolus variegatus – Ammobates syriacus*, min = 60. The time and memory estimates are for full RelTime-JA analysis. Normalized Robinson-Foulds (RF) distance (Robinson and Foulds, 1981) between the published and RelTime-JA timetrees. *References: (1) Álvarez-Carretero et al. (2022), (2) Ran et al. (2018), (3) Sann et al. (2018), (4) Pessoa-Filho et al. (2017), (5) Johnson et al. (2018), (6) Kuntner et al. (2019). All phylogenomic datasets are available at https://doi.org/10.6084/m9.figshare.22114943.

Supplementary Table S3. Summary of node supports/posterior probabilities and RF distances from simulated data analysis by applying the RelTime-JA with standard bootstrap method.

Dataset / no. of sites	ML normalized RF distance	BEAST2-JA mean node probability	BEAST2-JA normalized RF distance	MrBayes-JA mean node probability	MrBayes-JA normalized RF distance	RelTime-JA mean node support	RelTime-JA normalized RF distance
309s	0.21	0.81	0.27	0.89	0.27	0.71	0.30
450s	0.27	0.81	0.21	0.96	0.29	0.74	0.30
537s	0.08	0.86	0.08	0.91	0.15	0.82	0.11
782s	0.21	0.89	0.15	0.92	0.17	0.84	0.15
1073s	0.13	0.92	0.13	0.94	0.13	0.87	0.06
1523s	0.04	0.95	0.06	0.96	0.06	0.89	0.02
2116s	0.10	0.93	0.10	0.96	0.08	0.93	0.15
3100s	0.06	0.96	0.04	0.99	0.04	0.95	0.04
4070s	0.00	0.97	0.00	0.97	0.00	0.96	0.00
7002s	0.13	0.99	0.02	0.99	0.02	0.98	0.02
9539s	0.04	0.97	0.02	0.99	0.04	0.94	0.02

The normalized RF distances were calculated by comparing the model timetree with the inferred phylogenies. The simulated datasets and model timetree are available at https://doi.org/10.6084/m9.figshare.22114943.

References

Álvarez-Carretero, S., Tamuri, A. U., Battini, M., Nascimento, F. F., Carlisle, E., Asher, R. J., et al. (2022). A species-level timeline of mammal evolution integrating phylogenomic data. *Nature* 602, 263–267. doi: 10.1038/s41586-021-04341-1.

Johnson, K. P., Dietrich, C. H., Friedrich, F., Beutel, R. G., Wipfler, B., Peters, R. S., et al. (2018). Phylogenomics and the evolution of hemipteroid insects. *Proc. Natl. Acad. Sci. U. S. A.* 115, 12775–12780. doi: 10.1073/pnas.1815820115.

Kuntner, M., Hamilton, C. A., Cheng, R. C., Gregorič, M., Lupše, N., Lokovšek, T., et al. (2019). Golden Orbweavers Ignore Biological Rules: Phylogenomic and Comparative Analyses Unravel a Complex Evolution of Sexual Size Dimorphism. *Syst. Biol.* 68, 555–572. doi: 10.1093/sysbio/syy082.

Pessoa-Filho, M., Martins, A. M., and Ferreira, M. E. (2017). Molecular dating of phylogenetic divergence between Urochloa species based on complete chloroplast genomes. *BMC Genomics* 18, 1–14. doi: 10.1186/s12864-017-3904-2.

Ran, J. H., Shen, T. T., Wu, H., Gong, X., and Wang, X. Q. (2018). Phylogeny and evolutionary history of Pinaceae updated by transcriptomic analysis. *Mol. Phylogenet. Evol.* 129, 106–116. doi: 10.1016/j.ympev.2018.08.011.

Robinson, D. F., and Foulds, L. R. (1981). Comparison of Phylogenetic trees. *Math. Biosci.* 53, 131–141. doi: 10.1016/0025-5564(81)90043-2.

Sann, M., Niehuis, O., Peters, R. S., Mayer, C., Kozlov, A., Podsiadlowski, L., et al. (2018). Phylogenomic analysis of Apoidea sheds new light on the sister group of bees. *BMC Evol. Biol.* 18, 1–15. doi: 10.1186/s12862-018-1155-8.
